# PoDCall: positive droplet calling and normalization of droplet digital PCR DNA methylation data

**DOI:** 10.1093/bioinformatics/btac766

**Published:** 2022-11-30

**Authors:** Marine Jeanmougin, Hans Petter Brodal, Heidi Dietrichson Pharo, Hege Marie Vedeld, Guro Elisabeth Lind

**Affiliations:** Department of Molecular Oncology, Institute for Cancer Research, Oslo University Hospital, The Norwegian Radium Hospital, Oslo 0379, Norway; Department of Molecular Oncology, Institute for Cancer Research, Oslo University Hospital, The Norwegian Radium Hospital, Oslo 0379, Norway; Department of Molecular Oncology, Institute for Cancer Research, Oslo University Hospital, The Norwegian Radium Hospital, Oslo 0379, Norway; Department of Biosciences, The Faculty of Mathematics and Natural Sciences, University of Oslo, Oslo 0371, Norway; Department of Molecular Oncology, Institute for Cancer Research, Oslo University Hospital, The Norwegian Radium Hospital, Oslo 0379, Norway; Department of Molecular Oncology, Institute for Cancer Research, Oslo University Hospital, The Norwegian Radium Hospital, Oslo 0379, Norway; Department of Biosciences, The Faculty of Mathematics and Natural Sciences, University of Oslo, Oslo 0371, Norway

## Abstract

**Motivation:**

Droplet digital PCR (ddPCR) holds great promises for investigating DNA methylation with high sensitivity. Yet, the lack of methods for analyzing ddPCR DNA methylation data has resulted in users processing the data manually at the expense of standardization.

**Results:**

PoDCall is an R package performing automated calling of positive droplets, quantification and normalization of methylation levels in ddPCR experiments. A Shiny application provides users with an intuitive and interactive interface to access PoDCall functionalities.

**Availability and implementation:**

The PoDCall R package is freely available on Bioconductor at https://bioconductor.org/packages/PoDCall/. The Shiny application can be executed from the R console using the wrapper function PoDCall::podcallShiny().

**Supplementary information:**

Supplementary data are available at *Bioinformatics* online.

## 1 Introduction

Droplet digital PCR (ddPCR) provides researchers with an attractive solution for absolute DNA quantification. The method has been successfully used for detection of genetic variants, including in liquid biopsies where it shows high sensitivity (Pekin *et al.*, 2011). Analyses of copy number variations or investigations of gene expression are also common applications of ddPCR (Boettger *et al.*, 2012; Taylor *et al.*, 2017). Although ddPCR holds great promises for DNA methylation analyses, its use is held back by the lack of standardized approaches for data processing.

The ddPCR technology consists of partitioning samples into thousands of individual droplets, in which an independent PCR reaction is carried out. The assays used for detection comprise primers and fluorescent-labeled probes. After the amplification reactions are complete, the fluorescence signal of each droplet is measured. Droplets containing the target of interest typically cluster in a ‘positive’ cloud, with higher fluorescence amplitude values compared to droplets that are ‘negative’ for the target. QuantaSoft™, the default software coupled with the ddPCR™ BioRad system (https://www.bio-rad.com/digitalpcr), is commonly used for droplet calling and data interpretation but can fail setting accurate thresholds. A contributing factor is the presence of droplets occurring between the negative and positive clouds, a phenomenon known as ‘rain’. While rain can be significantly reduced by optimizing assays in many cases, it is more challenging in methylation experiments due to the specific requirements of probe and primer design. Destroyed droplets, with extremely low amplitude values, or shifts in the negative baseline distribution across wells can also be observed and affect the performances of QuantaSoft. A few alternative solutions have emerged for droplet calling, including heuristic approaches, such as k-means clustering (Chiu *et al.*, 2017; Jones *et al.*, 2014) or model-based clustering (Attali *et al.*, 2016; Jacobs *et al.*, 2017; Trypsteen *et al.*, 2015). However, to the best of our knowledge, none of these methods have been extensively tested on DNA methylation data, nor provided ways to normalize concentrations by accounting for differences in template amount and potential chromosomal aberrations.

To fill this gap, we introduce PoDCall, an R package and shiny graphical user interface (GUI) for model-based positive droplet calling of ddPCR DNA methylation data. PoDCall performs quantification of DNA methylation levels and returns raw concentration values. Normalized concentrations are also accessible if an internal control is provided, either as a single gene (e.g. *ACTB, MYOD1* and *COL2A1*) or as the 4Plex, a control previously established by our group that amplifies four single copy genes located on different chromosomes (Pharo *et al.*, 2018).

## 2 Materials and methods

### 2.1 Input data

PoDCall uses well-specific .csv files from the Bio-Rad’s ddPCR system as input data and enables uploading one or multiple files simultaneously. Each .csv file consists of a two-column data frame containing fluorescence amplitude values for the two channels (possibly, a target and a control), and one row for each droplet in the well. As encouraged by the digital MIQE guidelines (The dMIQE Group and Huggett, 2020), a commercially available *in vitro* methylated DNA (IVD; 100% methylated DNA) should be used as a methylation-positive control. The inclusion of an internal control is also strongly recommended for normalization purposes; we suggest using the methylation-specific 4Plex control rather than a single locus control as gene panels have been demonstrated to increase the precision of ddPCR analyses (Coulter, 2018; Pharo *et al.*, 2018). An optional sample sheet exported from QuantaSoft and including columns with well name (‘Well’), sample name (‘Sample’), type of target - e.g. target gene or control (‘TargetType’), and name of target gene (‘Target’), can also be provided.

### 2.2 Positive droplet calling

Droplet calling consists in finding a threshold on amplitude values to classify droplets. Droplets with amplitude values > threshold are assumed to contain the target molecule and are labeled ‘positive’. PoDCall performs droplet calling stepwise, as illustrated in Supplementary Figure S1 and detailed in Supplementary Materials and Methods. The full workflow runs in R using the wrapper function PoDCall::podcallDdpcr().

### 2.3 Quantification and normalization of methylation levels

The positive droplets are used to estimate the target concentration in the sample using a Poisson distribution as described in the Instruction Manual of QuantaSoft™ Analysis Pro Software (https://www.bio-rad.com/webroot/web/pdf/lsr/literature/QuantaSoft-Analysis-Pro-v1.0-Manual.pdf). If a single gene is provided as internal control, normalized concentrations are calculated as percentages: $\frac{\left[\mathrm{channel}\;1\;\mathrm{concentration}\;(\mathrm{target}\;\mathrm{gene})\right]}{\left[\mathrm{channel}\;2\;\mathrm{concentration}\;(\mathrm{internal}\;\mathrm{control})\right]}*\;c,$ with c = 100. Since the 4Plex is a four-assay panel, when it is used for normalization we set c = 400, to be in the same range of values as single gene normalized concentrations. In the absence of an internal control in channel 2, the argument ‘ch2’ of the wrapper function podcallDdpcr() must be set to ‘FALSE’.

### 2.4 Output

Results are returned as a table, optionally written to a .csv file. Plots are saved as .pdf files in the results directory if argument ‘plots’=TRUE.

### 2.5 Shiny GUI

The PoDCall package includes a user interface, written using the Shiny package 1.6.0. The interface allows users to perform droplet calling and normalization in an interactive environment, where thresholds can also be visually inspected and manually corrected. The application runs locally using the PoDCall::podcallShiny() function.

## 3 Results

**Fig. 1.**
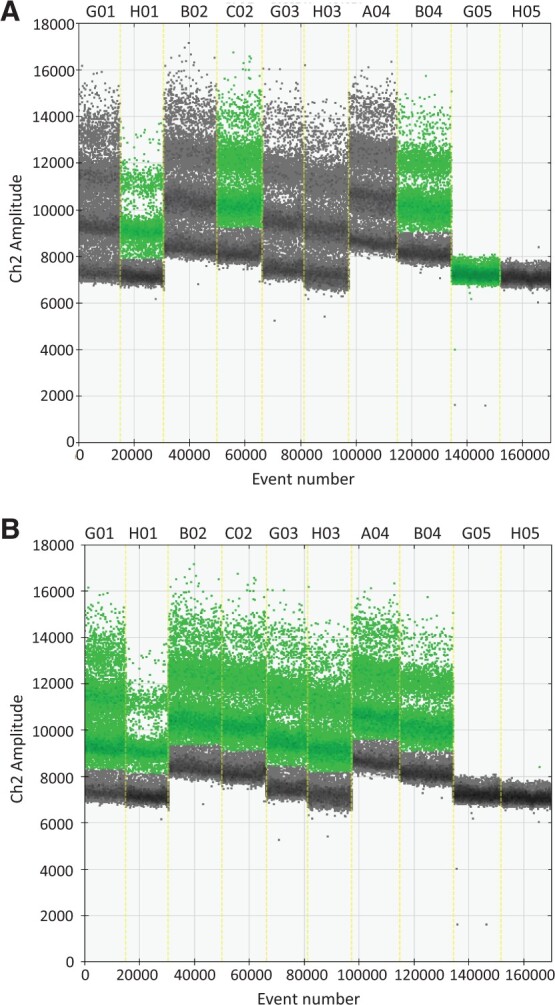
Comparison of positive droplet calling using the QuantaSoft software *versus* PoDCall. (**A**) Calling of positive droplets (in green) using QuantaSoft. (**B**) Calling of positive droplets (in green) using PoDCall. Wells G01 to B04 include colorectal cancer cell line samples (G01, Colo320; H01: Colo678; B02: EB; C02: FRI; G03: RKO; H03: SW1116; A04: SW1463; B04: SW403), G05 is non-bisulfite-treated DNA and H05 a no-template control (NTC).

A ddPCR DNA methylation dataset and its sample sheet are provided as examples and available in the package (see description in Supplementary Materials and Methods). PoDCall has been extensively evaluated on DNA methylation data across various sample types including cancer cell lines, tissues and liquid biopsies, with R version 4.1. Figure 1 illustrates some limitations encountered with QuantaSoft and overcome by PoDCall; while QuantaSoft fails to call positive droplets in over half of the wells (Fig. 1A) and shows sensitivity to negative outliers (well G05), PoDCall demonstrates its ability to robustly classify droplets across all samples (Fig. 1B).

## 4 Conclusion

We developed PoDCall, a user-friendly solution for automated droplet calling of ddPCR DNA methylation data, combining a model-based approach with an outlier detection strategy. PoDCall provides raw DNA methylation concentration values and also gives the possibility to use either the 4Plex or single markers as internal controls for normalization purposes. It contributes to increasing the robustness of ddPCR DNA methylation analyses and enables the detection of targets with low concentrations, including in liquid biopsies.

## Supplementary Material

btac766_Supplementary_Data
