# Supplementary material for: PoDCall: positive droplet calling and normalization of droplet digital PCR DNA methylation data
Source: Bioinformatics. 2022 Nov 30;39(1):btac766. doi: 10.1093/bioinformatics/btac766 (PMC9825742; doi:10.1093/bioinformatics/btac766)
Supplement: btac766_Supplementary_Data [file btac766_supplementary_data.zip › SupplementaryMaterialAndMethods_Jeanmougin.pdf]

# Supplementary Material and Methods

## 1. ddPCR methylation dataset

A set of amplitude files from ddPCR methylation analyses of three colorectal cancer cell lines (SW1463, SW403 and SW480), a positive control (IVD) and a negative control (NTC; non-template control), is provided in the package. The target gene in channel 1 is vimentin (*VIM*), and the internal control in channel 2 is the 4Plex. Assays were designed where the target gene was labelled with FAM (channel 1) and the internal control with VIC (channel 2). DdPCR methylation data were generated on the Bio-Rad QX200™ Droplet Digital™ PCR System.

## 2. Implementation

The algorithm was implemented as an R package (R version >= 4.1). The full workflow runs either in R with the wrapper `podcallDdpcr()` function or using the shiny GUI.

## 3. Positive droplet calling

Droplet calling consists in finding a threshold of amplitude values to classify droplets (Figure S1); droplets with amplitude values > threshold being labeled positive.

**Step 1 Reference calling -** Using a reference well, ideally a methylation-positive control - *e.g.* IVD, PoDCall first tests for unimodality of the amplitude value distribution (null hypothesis, $H_0$) *via* the Hartigan's dip test (see Figure S1-Step1).

(1) If $H_0$ is rejected, a likelihood ratio test (LRT) is performed to identify the number of Gaussian mixture components in the amplitude value distribution (package `mclust`). Bootstrap resampling is used to compute significance of the LRT as implemented in the `mclustBootstrapLRT()` function. The number of bootstrap replications B is set to 200 but can be adjusted by users. The reference threshold $thr_{ref}$ is defined as the mean between the first and second components. We demonstrated in Table S1 that normalised concentrations are very similar in the range B=50-2000 on test data, with computational time<2min for B=10-200.

(2) If $H_0$ is not rejected, we test for the presence of droplets with amplitudes values > UQ + Q*IQR, with UQ and IQR the upper quartile and interquartile range of the amplitude value distribution, respectively, and Q a constant defined by the user and set to 9 by default. Testing for outliers allows to overcome some limitations of the Hartigan's dip test, which can be too conservative when the second mode contains too few values and fails to reject the null hypothesis. The reference threshold

$thr_{ref}$ is then set as the maximum amplitude value once potential positive outliers are discarded (see Figure S2 for examples).

**Step 2 Well-adjusted calling -** To account for potential shifts in the negative droplet distribution as illustrated on Figure 1, $thr_{ref}$ is adjusted for each individual well (see Figure S1-Step2).
