# Supplementary material for: PoDCall: positive droplet calling and normalization of droplet digital PCR DNA methylation data
Source: Bioinformatics. 2022 Nov 30;39(1):btac766. doi: 10.1093/bioinformatics/btac766 (PMC9825742; doi:10.1093/bioinformatics/btac766)
Supplement: btac766_Supplementary_Data [file btac766_supplementary_data.zip › TableS1_Jeanmougin.pdf]

Table S1 - Normalised concentration values according to number of bootstrap samples (B). Wells A04, B04 and D04 contain colorectal cancer cell line samples, SW1463, SW403 and SW480; well D05 is a positive control (IVD; in vitro methylated DNA) and well H05 is a negative control (NTC; non-template control).

B	Well					Computational time (min)
	A04	B04	D04	D05	H05	
**10**	No DNA	No DNA	No DNA	No DNA	No DNA	1’22
**20**	45.8	24.6	1.14	71.6	0	1’28
**50**	47.6	26.7	1.18	75.0	0	1’34
**100**	47.8	26.9	1.18	75.5	0	1’39
**200**	47.7	26.7	1.18	75.1	0	1’50
**400**	47.5	26.6	1.18	74.9	0	2’14
**1000**	47.6	26.7	1.18	75.0	0	3’20
**2000**	47.7	26.7	1.18	75.2	0	5’29
