# Supplementary figures and images for: PoDCall: positive droplet calling and normalization of droplet digital PCR DNA methylation data
Source: Bioinformatics. 2022 Nov 30;39(1):btac766. doi: 10.1093/bioinformatics/btac766 (PMC9825742; doi:10.1093/bioinformatics/btac766)

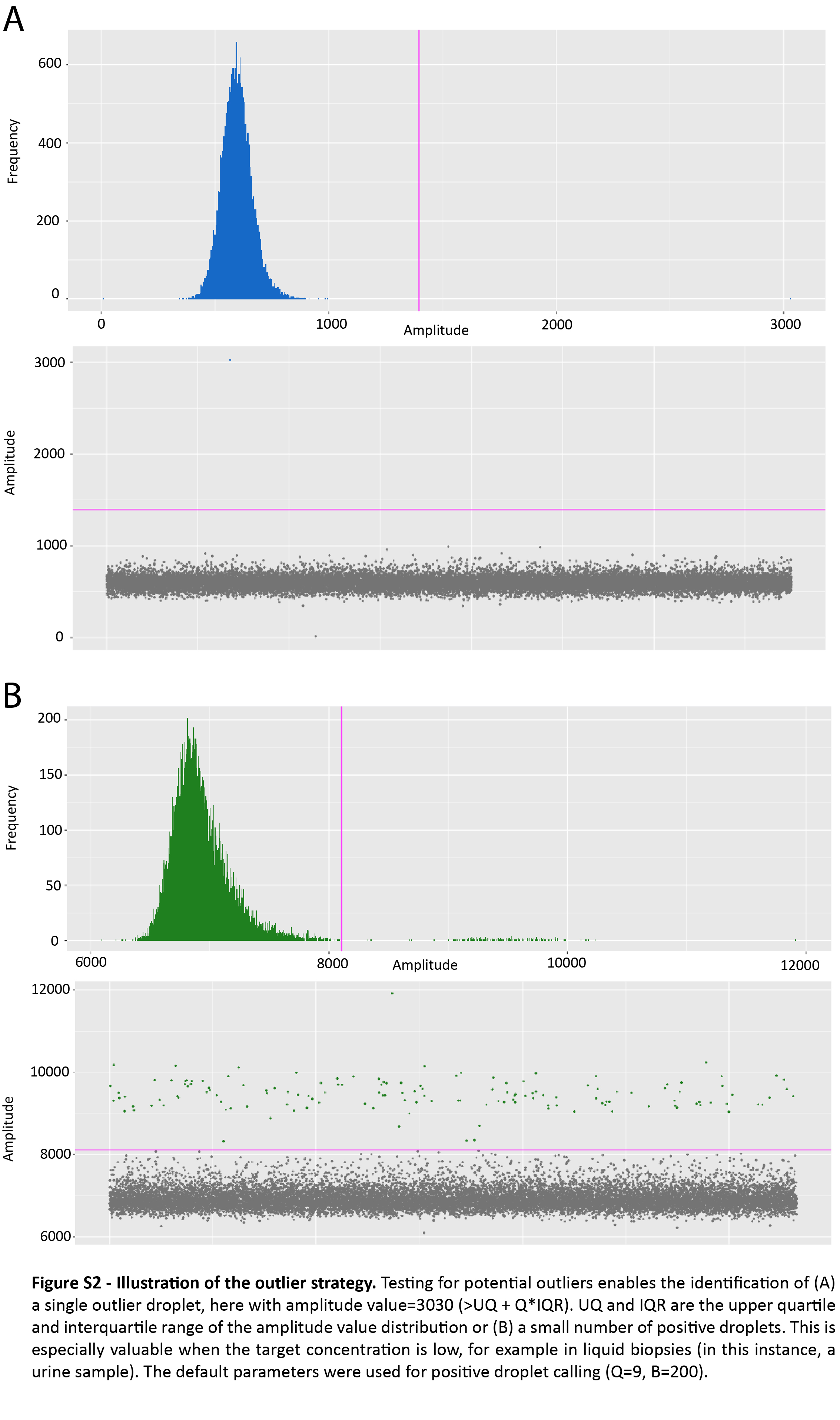

Supplement: btac766_Supplementary_Data [file btac766_supplementary_data.zip › FigureS2.jpg]

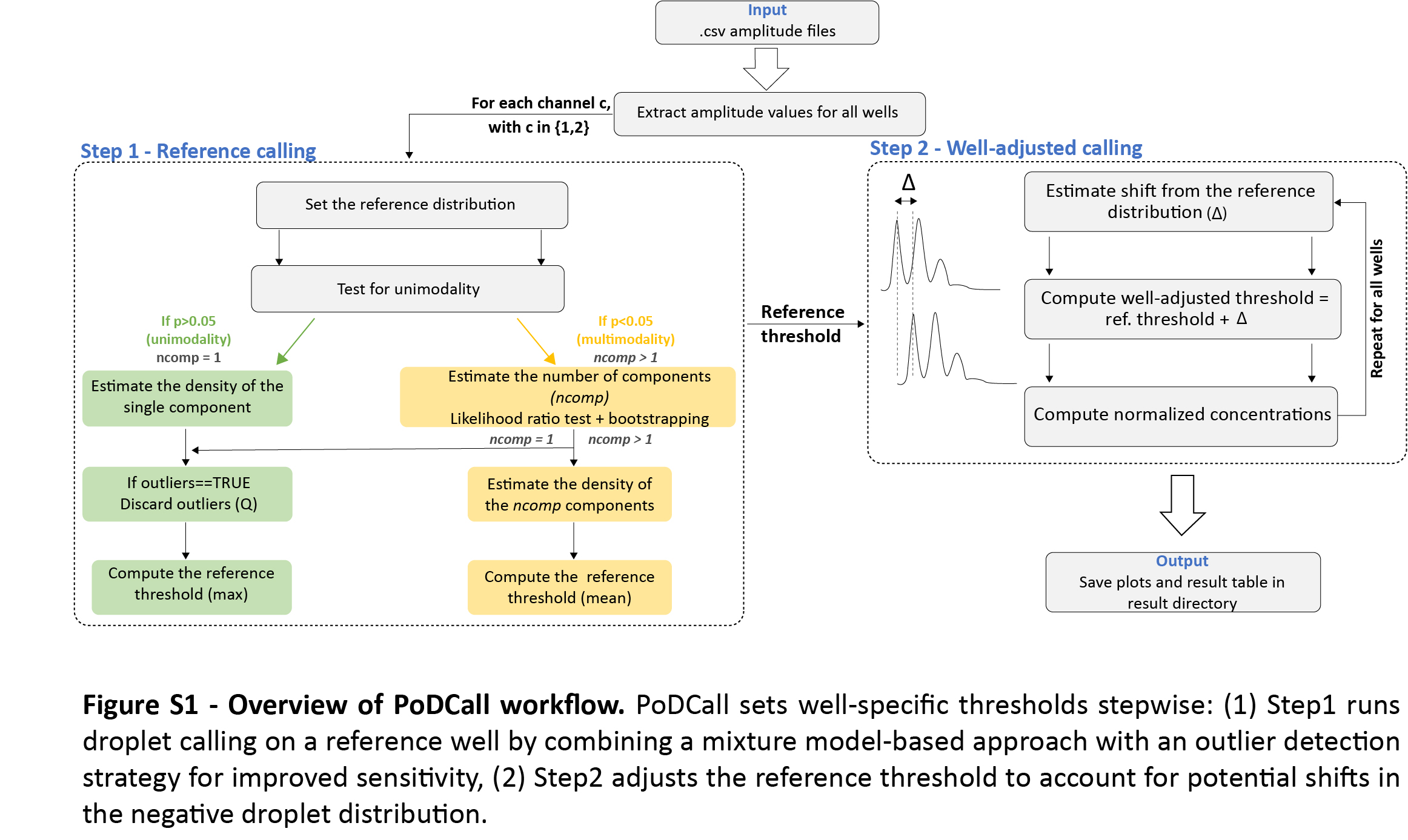

Supplement: btac766_Supplementary_Data [file btac766_supplementary_data.zip › FigureS1.jpg]
